# Higher incidence of hip fracture in newly diagnosed schizophrenic patients in Taiwan

**DOI:** 10.3402/ljm.v8i0.20911

**Published:** 2013-04-17

**Authors:** Shih-Wei Lai, Cheng-Li Lin, Kuan-Fu Liao

**Affiliations:** 1 School of Medicine, China Medical University, Taichung, Taiwan; Department of Family Medicine, China Medical University Hospital, Taichung, Taiwan; 2 Department of Public Health, China Medical University, Taichung, Taiwan; Management Office for Health Data, China Medical University Hospital, Taichung, Taiwan; 3 Graduate Institute of Integrated Medicine, China Medical University, Taichung, Taiwan; Department of Internal Medicine, Taichung Tzu Chi General Hospital, Taichung, Taiwan; Department of Health Care Administration, Central Taiwan University of Science and Technology, Taichung, Taiwan

Hip fracture is a major public health concern due to its poor outcome and serious socioeconomic burden in older people (1). Evidence has shown that many factors are related to increased risk of hip fracture, but psychiatric diseases are not confirmatory (2, 3). A case-control study by Howard et al. in the United Kingdom has shown that schizophrenia is associated with increased risk of hip fracture in univariate analysis (odds ratio [OR]=1.73, 95% CI=1.32–2.28), but association is no longer found in multivariate analysis (3). To date, no clinical study has focused on the relationship between schizophrenia and the risk of hip fracture in older people in Taiwan. In order to clarify this issue, we used the database from the Taiwan National Health Insurance program to conduct this population-based cohort study. The details of the insurance program can be found in previous studies (4–6).

In this study, we randomly selected 445 subjects aged 65 years or older with newly diagnosed schizophrenia as the case group based on the diagnosis codes of the International Classification of Diseases, 9th Revision, Clinical Modification (ICD-9 codes 295 and V11.0, 210 men and 235 women, mean age [standard deviation]=75.2 [6.9] years) and 1,780 subjects aged 65 years or older without schizophrenia as the comparison group (840 men and 940 women, mean age [standard deviation]=74.2 [7.1] years), from 2000 to 2010. Both groups were matched for gender, age and year of diagnosis. We measured the incidence of hip fracture (ICD-9 codes 820) until either hip fracture was diagnosed or until 31 December 2010. To reduce the biased results, subjects with hip fracture diagnosed before entering this cohort were excluded.

The schizophrenia group had a significantly higher incidence of hip fracture than did the non-schizophrenia group (20.3 vs. 10.6 per 1,000 person-years, incidence rate ratio [IRR]=1.91, 95% CI=1.49–2.44). The subanalysis stratified by gender and age group also displayed that the incidence rates of hip fracture were all higher in the schizophrenia group than that in the non-schizophrenia group, with statistical significance. Men with schizophrenia had a higher risk of hip fracture than did women with schizophrenia (IRR 3.26 vs. 1.42). Subjects with schizophrenia aged 75–84 had the highest incidence than other subgroups (incidence rate=35.4 per 1,000 person-years), but subjects with schizophrenia aged 65–74 had a higher risk of hip fracture (IRR=2.14, 95% CI=1.51–3.02). The risk of hip fracture was higher among subjects with a duration of diagnosing schizophrenia of less than 2 years (IRR=2.82, 95% CI=2.16–3.69), as compared to those with a duration ≥2 years (IRR=1.54, 95% CI=1.14–2.07) (Table 1).

**Table 1:** Incidence rates of hip fracture for schizophrenia group and non-schizophrenia group

	Non-schizophrenia				Schizophrenia				
			
	N	Case	Person-years	Incidence rate	N	Case	Person-years	Incidence rate	Incidence rate ratio (95% CI)†
All	1,780	113	10,640	10.6	445	42	2,072	20.3	1.91 (1.49, 2.44)
Gender									
Men	840	30	4,770	6.29	210	17	830	20.5	3.26 (2.27, 4.67)
Women	940	83	5,870	14.1	235	25	1,242	20.1	1.42 (1.02, 1.99)
Age group (year)									
65–74	956	37	6,611	5.60	239	16	1,338	12.0	2.14 (1.51, 3.02)
75–84	824	76	4,029	18.9	206	26	734	35.4	1.88 (1.34, 2.64)
Follow-up period									
<2 years	340	29	3,232	8.97	130	19	751	25.3	2.82 (2.16, 3.69)
≥2 years	1,440	84	7,408	11.3	315	23	1,321	17.4	1.54 (1.14, 2.07)

Incidence rate: per 1,000 person-years.

† Incidence rate ratio: schizophrenia vs. non-schizophrenia (95% CI).

A case-control study by Bolton et al. in Canada has shown that schizophrenia is associated with increased risk of osteoporotic fractures after being adjusted for covariates (OR=1.61, 95% CI=1.27–2.04) but not focusing on hip fracture (7). In this study, patients with schizophrenia have a 1.9-fold increased risk of hip fracture. In further analysis, we also found that the risk of hip fracture is 2.8-fold in patients with duration of less than 2 years after diagnosing schizophrenia, as compared to subjects without schizophrenia. This means that more efforts should be made to prevent falls in older people with schizophrenia, particularly in the first 2 years of diagnosis. Due to inconsistent results from the aforementioned studies, additional studies are needed to determine the relationship between schizophrenia and hip fracture incidence in older people.

*Shih-Wei Lai* School of Medicine China Medical University Taichung, Taiwan Department of Family Medicine China Medical University Hospital Taichung, Taiwan*Cheng-Li Lin* Department of Public Health China Medical University Taichung, Taiwan Management Office for Health Data China Medical University Hospital Taichung, Taiwan *Kuan-Fu Liao* Graduate Institute of Integrated Medicine China Medical University Taichung, Taiwan Department of Internal Medicine Taichung Tzu Chi General Hospital Taichung, Taiwan Department of Health Care Administration Central Taiwan University of Science and Technology Taichung, Taiwan E-mail: kuanfuliao@yahoo.com.tw
